# Investigation on Advanced Non-Small-Cell Lung Cancer among Elderly Patients Treated with Chinese Herbal Medicine versus Chemotherapy: A Pooled Analysis of Individual Data

**DOI:** 10.1155/2019/1898345

**Published:** 2019-01-02

**Authors:** Lingling Sun, Wan Sze Yim, Paul Fahey, Shutang Wang, Xiaoshu Zhu, Jing Qiao, Hezheng Lai, Lizhu Lin

**Affiliations:** ^1^Integrative Cancer Centre, Guangzhou University of Chinese Medicine First Affiliated Hospital, Guangzhou, Guangdong, China; ^2^School of Chinese Medicine, Chinese University of Hong Kong, Hong Kong; ^3^School of Science and Health, Western Sydney University, Campbelltown, NSW, Australia; ^4^Translational Health Research Institute, Western Sydney University, Campbelltown, NSW, Australia; ^5^National Institute of Complementary Medicine (NICM), Western Sydney University, Campbelltown, NSW, Australia

## Abstract

**Purpose:**

Many patients with advanced non-small-cell lung cancer (NSCLC) seek help from Chinese herbal medicine (CHM). The purpose of this study was to investigate the survival between CHM and chemotherapy (CT) treatment of patients aged ≥60 years with advanced Epidermal Growth Factor Receptor (EGFR) wild type NSCLC and Karnofsky Performance Status (KPS) ≥ 60.

**Methods:**

We extracted individual data of all eligible patients from 1 randomized control trial and 2 cohort studies and performed a pooled analysis. Survival outcomes of patients were compared between CHM group and CT group using Cox regression model stratified for study.

**Results:**

A total of 486 patients were included in the study, including 262 patients in the CHM group and 224 patients in the CT group. The median overall survival time was 10.9 (95% confidence intervals [CI]: 8.9-13.0) months in CHM group and 9.8 (95% CI: 8.1-11.5) days in CT group (p=0.592). The adjusted hazard ratio (HR) and 95% CI for CHM compared to CT are 0.98 (0.87, 1.10, p=0.751) in the stratified Cox regression model. Stratified analysis showed a trend that previously treated elderly patients with EGFR wild type advanced NSCLC probably gain greater survival benefit from CHM (adjusted HR:0.83, 95% CI: 0.68-1.01, p=0.063).

**Conclusions:**

There might be no significant difference in survival for elderly patients with advanced EGFR wild type NSCLC between the CHM and CT groups in the current study. And previously treated elderly patients with advanced NSCLC probably receive greater benefit from CHM. However, limited by the design and unpreplanned study hypothesis, the results must be confirmed by randomized control trial before making a conclusion.

## 1. Introduction

Lung cancer is the most frequently diagnosed cancer. An estimated 1.8 million new lung cancer cases occurred in 2012, accounting for about 13% of total cancer diagnoses [1]. It is also the most common cause of death from cancer worldwide, responsible for nearly one-fifth of all cancer deaths (1.59 million deaths, 19.4% of the total) [2]. Non-small-cell lung cancer (NSCLC) is the most common type of lung cancer, accounting for about 80% of all cases [3, 4]. More than half of the NSCLC cases are diagnosed at an advanced stage (stages III and IV) [5], and 63% of cases are 65 years of age or older. Demographics that are shifting toward an older population suggest that oncologists will be seeing more elderly patients with NSCLC in years to come [6, 7].

In patients with NSCLC, only 20-25% of the cases harbor treatable driver mutations, such as Epidermal Growth Factor Receptor (EGFR) mutation, for which tyrosine kinase inhibitor can be used. 75%-80% of NSCLC cases are EGFR wild type and cannot gain any benefit from target therapy [8]. Bevacizumab, a monoclonal antibody which targets vascular endothelial growth factor, can be applied to EGFR wild-type NSCLC, but does not improve overall survival for patients over the age of 65 [9]. To date, chemotherapy (CT) remains the standard of care for older patients with good functional status [10, 11]. However, a treatment dilemma of how to choose the use of CT in the care of older patients with advanced NSCLC is very common in the clinical setting [12, 13], given CT can prolong survival but can also significantly increase the incidence of side effects [14, 15]. Due to the fear of severe adverse reactions, many patients are being undertreated through discontinuing or not receiving CT [16]. An effective and safe therapy for elderly patients with advanced NSCLC was a largely unmet treatment need until recently.

Chinese herbal medicine (CHM) is reported as a safe alternative therapy with many roles in improving symptoms, such as reducing cancer-related fatigue, improving gastrointestinal side effects, protecting liver function, and even ameliorating bone marrow suppression [17, 18]. And CHM, as an adjunct to the conventional antitumor therapy, may improve overall survival of lung cancer patients [19, 20]. Our previous studies also showed that CHM plus chemotherapy might improve overall survival compared chemotherapy alone [21, 22]. As a result of CHM's advantage in reducing side effects and its potential role in prolonging survival, in China many elderly patients with advanced NSCLC, who discontinue CT, swap to CHM. However, the survival effect of CHM alone for this population compared to CT alone is poorly studied.

We have developed a standard CHM formulation called Yiqi Chutan Fang for treating NSCLC. We have conducted 3 clinical research studies to see if this CHM formulation (Yiqi Chutan Fang is the basic formula) had any a positive effect on advanced NSCLC [21–24], and we have performed laboratory research to explore the effect of the formula on tumor growth and metastasis in the past 16 years [25, 26]. These studies indicated this formula is a safe therapy and has a positive role in inhibiting tumor progression. One of the studies indicated that compared to patients received chemotherapy alone, patients received CHM alone has a lower incidence of fatigue, gastrointestinal side effects, bone marrow suppression and liver damage [21]. Each of the three clinical studies included EGFR wild type or untested patients aged ≥ 60 years with advanced NSCLC and Karnofsky Performance Status (KPS) ≥ 60 who received CHM or CT alone. The aim of the current paper is to investigate the impact of CHM versus CT on the survival of these patients and subgroups from that population by performing pooled analysis of individual data from these three studies.

## 2. Method

### 2.1. Research Design and Patients

We extracted individual data of patients aged 60 or older with advanced EGFR wild-type NSCLC and KPS ≥ 60 from our three clinical research studies and conducted a pooled analysis to compare the survival between CHM users and CT users. Patients included in the present analysis should meet the criteria as follows:

- Aged 60 and older.
- KPS ≥ 60.
- Stage III or IV according to Union for International Cancer Control (UICC) of the time.
- EGFR wild type or untested.
- Received CHM or CT only. Patients who received EGFR tyrosine kinase inhibitors in the follow-up period were excluded in order to reduce bias. Patients who received best supportive care only were also excluded.

### 2.2. Three Clinical Studies

We performed three multicenter clinical studies to investigate the effectiveness of CHM on advanced NSCLC including one randomized controlled trial (RCT) and two cohort clinical studies during 2001 to 2016 [21–23]. Key characteristics of these three studies are shown in Table 1.

All patients had advanced NSCLC and EGFR wild type or untested. All patients received treatment immediately after entry into the studies. The CHM treatment received is Yiqi Chutan Fang, with minor modification according to changes in patient with symptoms. CT and best supportive care (BSC) is the conventional treatment in clinical practice which followed the guidelines of National Comprehensive Cancer Network of the time. Overall survival time is the primary outcome. There was no overlapped enrollment among these three studies; that is, no single patient was included or assessed in more than one study.

### 2.3. Clinical Variables and Outcome

The primary outcome variable of this study is survival time. Survival time was defined as the number of days from study entry to death from any cause. Where no death was recorded, survival time was censored at the latest follow-up.

The primary predictor variable is treatment received, CHM, or CT. The main Chinese herbal formula, Yiqi Chutan Fang, the patients received consisted of Ban Xia (pinellia ternate) 15 g, Xi Yang Shen (American ginseng) 30 g, Shan Ci Gu (pseudobulbus cremastrae seu pleiones) 30 g, and Zhe Bei Mu (bulbus fritillariae thunbergii) 15 g. Minor modifications on the herbs and the dosage were done according to symptom changes in each patient.

The chemotherapy regimen patients received included gemcitabine, docetaxel, pemetrexed, and vinorelbine, used as single agent or in combination with platinum-based drugs. Personalized dosage was given based on patient's physical condition. The minimum dosage given was at least 60% of the standard dosage. Potential confounder variables available in all three studies and included in the present study are as follows:

- Demographic information (age; gender).
- Smoking history; smoker was defined as patients with present or past smoking behavior. Nonsmoker was defined as patients who never smoked.
- Tumor stage (Stages III and IV).
- Tumor location classification (central type; peripheral type).
- Tumor pathological type (non-squamous-cell carcinoma (non-SQCC); squamous cell carcinoma (SQCC)).
- Previously treated (No; Yes).
- KPS.

### 2.4. Sample Size Considerations

We did not conduct sample size calculations as data collection was already completed. Instead, we focused on the confidence intervals according to Smith and Bates' advice [27].

### 2.5. Statistical Analysis

We pooled the eligible records from each of the three studies into a single data set. The baseline characteristics of patients treated with CT alone and those treated with CHM are presented as counts and percentages for categorical variables, means, and standard deviations for numeric variables. We used Pearson's Chi-square test and independent samples t-tests to test the statistical significance of the differences between groups.

We also estimated the follow-up period by a reverse Kaplan-Meier method [28] and presented it as median and 95% confidence intervals (CI). We also presented the survival times within each treatment group as a naive Kaplan-Meier plot, which assumed there are no differences between these three studies. Then we used a stratified log-rank test to test for the statistical significance of any difference in survival time between CHM and CT groups. This was followed up with analyses using a stratified Cox regression model approach [29] which allowed hazards to differ between the three component studies. Hazard ratios (HR) and associated 95% CI are presented for the difference in risk of mortality between treatment groups. As the cohort studies are not randomized we must adjust for all important differences between the CT and CHM groups which could confound patient survival times [30]. All potential predictor variables collected such as demographic factors (age, gender, and smoking status); tumor stage (III, IV); tumor location classification (central type; peripheral type); tumor pathological type (non-SQCC, SQCC); whether received previous treatment (No; Yes); KPS; treatment received (CHM or CT) were tested for their individual association with survival time using univariate stratified Cox models. The age variable was fitted as both a continuous and a dichotomous variable (<70 or ≥70). KPS was divided into ordinal categories (60, 70, ≥80). All potential confounders with p-values<0.2 and whether received previous treatment were included in a multivariable stratified Cox model. The demographic factors (age; gender) were also included in a multivariable stratified Cox model as sensitivity analysis.

We also used stratified multivariate Cox model to perform stratified analyses according to stage, smoking status, pathology, performance status, age, and previous treatment categories, in order to confirm the confounding effect of these factors. This analysis also could explore whether or not there are any subgroups of patients who could receive greater benefit from CHM treatment. The stratified model allows the baseline hazard to vary between groups, for example, allowing the hazard for squamous cell to differ from the hazard of non-squamous-cell cancers. The survival times of previously treated patients were also presented as a naive Kaplan-Meier plot.

Missing data occurred in the recording of tumor location and smoking status. We chose to not to impute the missing data as tumor location appeared to have no effect on survival and relatively few individuals had missing smoking status. The significance threshold was set at p-values <0.05 for all statistical tests in the study, unless otherwise stated. Statistical analyses were performed using Empower (R) (http://www.empowerstats.com, X&Y Solutions, Inc., Boston, MA).

## 3. Results

### 3.1. Patient Characteristics

**Figure 1:**
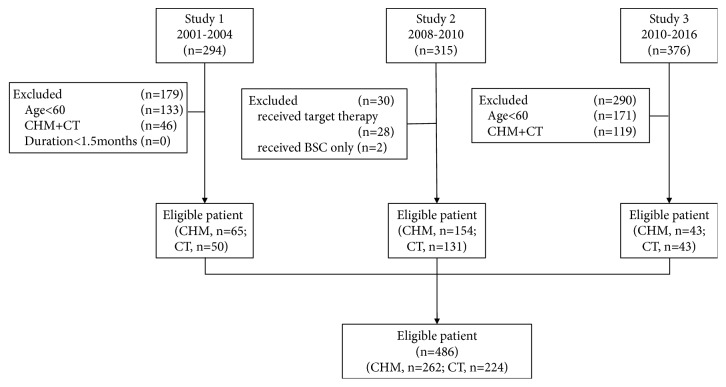
Flow diagram of eligible records extracted.

The process of data extraction was shown in Figure 1. A total of 486 patients were included in the analysis, in which 262(53.9%) patients received CHM alone and 224 (46.1%) patients received CT alone. The average age of patients was 68.8 ± 5.9 years, 297 (61.1%) people less than 70 years old, and 189 (38.9%) more than 70 years old. There were 335 (68.9%) male patients and 151 (31.1%) female patients. Approximately 90 percent of patients in CT group received CT for more than 4 courses.

The demographic information and clinical characteristics of the two groups are shown in Table 2. There was a difference of gender, age, and proportion with previous treatment between two groups. There were relatively more females, elderly patients more than 70 years old, and patients who previously received CT or radiotherapy in the CHM group than in the CT group.

### 3.2. Overall Survival

**Figure 2:**
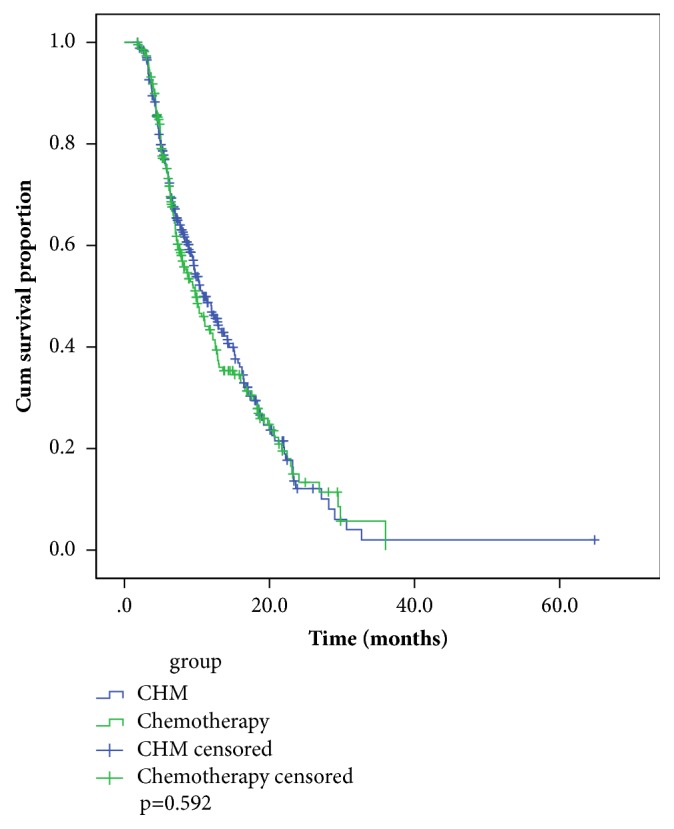
Kaplan-Meier survival curves for patients by treatment groups.

The median follow-up time for patients was 16.9 (95% CI:14.3-18.5) months. A total of 57 (11.7%) patients were lost to follow-up and 309 (63.6%) deaths occurred. The median overall survival time was 15.0 months (95% CI: 11.9-18.1) in CHM group and 18.3 months (95% CI:15.8-20.8) in the CT group (Figure 2). A stratified log-rank test showed that there is no significant difference in survival times between the two groups (p=0.592).

### 3.3. Mortality Risk

We used a stratified Cox model to compare survival times between CHM and CT patients with advanced NSCLC. The unadjusted stratified Cox model demonstrated that only smoking status, KPS, and stage showed any relationship with survival time of advanced NSCLC at the p<0.20 level. We found no statistically significant differences in survival between the CHM and CT groups in the univariate stratified Cox model (HR 0.97,95% CI:0.87-1.09, p=0.588) and in the multivariate model which adjusts for the different potential confounders: smoking status, KPS, stage, and previous treatment (HR 0.98, p=0.751, Table 3). Sensitivity analysis showed the result is stable when age and gender were also included in the stratified multivariate Cox model (HR:0.98, 95% CI:0.88-1.10, p=0.774). The 95% CI in the multivariate model indicated that the survival may be anything from 13% better to 10% worse in the CHM group (95% CI:0.87, 1.10, Table 3).

### 3.4. Stratified Analyses

In order to confirm the confounding effect on the result of the potential confounders, we performed subgroup analysis on pathology, performance status, age, smoking status, stage, and previous treatment categories. The unadjusted HR and adjusted HR are listed in Table 4. None of the results from the subgroup analysis display statistical significant differences in survival between the CHM and CT treatment groups.

**Figure 3:**
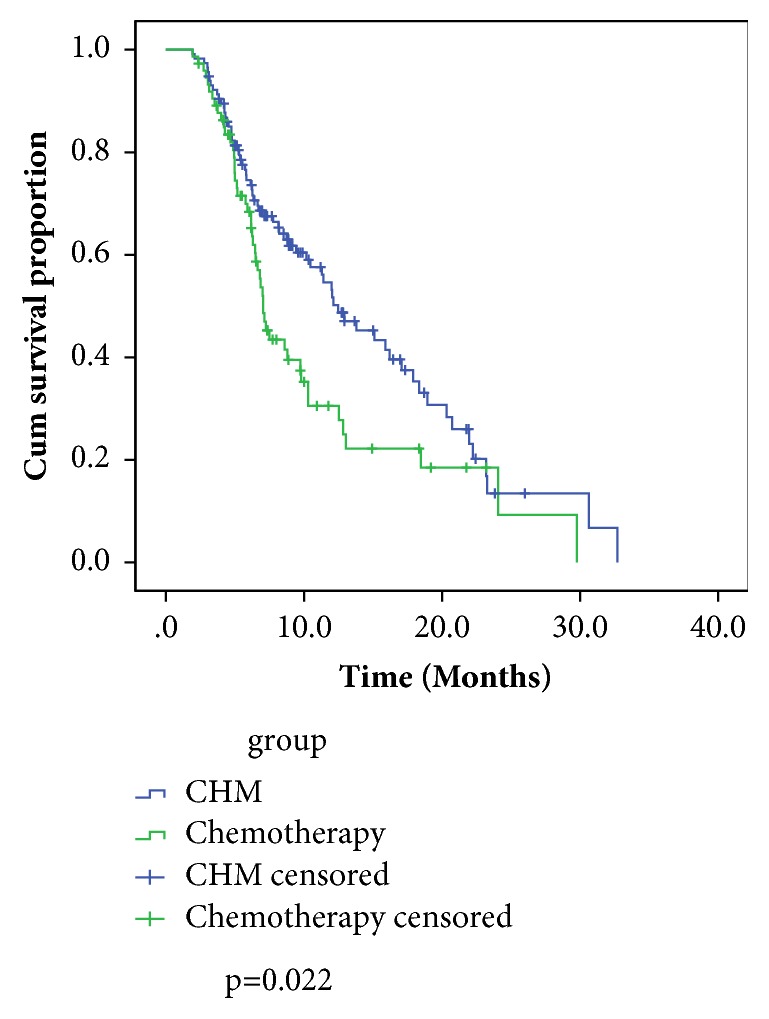
Kaplan-Meier survival curves for previously treated patients by treatment groups.

Although the subgroup analyses have smaller sample sizes and lower statistical power, we also consider the clinical importance of the difference in HRs between untreated patients and previously treated patients, which show differences in survival between CHM and CT groups, and might be of clinical interest. Patients those previously treated probably gain more survival benefit from CHM compared to CT (HR:0.83, 95% CI:0.68-1.01). The median survival of previously treated patients received CHM was 5.5 months longer than that of previously treated patients received CT (p=0.022, Figure 3).

## 4. Discussion

Based on the pooled analysis, it was found that while there is no evidence of any difference in survival of elderly patients with advanced NSCLC and KPS ≥ 60 between the CHM and CT groups, the 95% CI indicates that the survival may be anything from 13% better to 10% worse in the CHM group. And a trend also exists that previously treated elderly patients with EGFR wild type advanced NSCLC probably gain greater survival benefit from CHM. To our best knowledge, the present study is the largest study that explores the difference in survival of elderly patients with advanced NSCLC and KPS ≥ 60 between CHM and CT treatment.

The strength of the research is that the patients included in the study are strictly confined to patients with EGFR wild type or untested, advanced stage, and good performance. Thus, the confounding factors are greatly reduced. Another strength is that we have adjusted for possible confounding factors when using data from cohort studies. By extracting individual information of patients with the same characteristic and under the same treatment (either CHM or CT only) from three individual but related clinical research studies, the individual patient data pooled analysis provides high quality evidence with validity. Instead of simple pooled analysis, this is a redesign study which uses the previous data to solve a new problem.

The result that the survival may be anything from 13% better to 10% worse in the CHM group indicates the possibility of noninferior efficacy of CHM treatment in contrast to CT treatment, as the limit of the 95% CI for the hazard ratio of 0.8 to 1.2 was the acceptable margin in the most of noninferiority trials on oncology [31]. Under this circumstance, other issues such as frailty, side effects, and patient preference become relatively important considerations in deciding which is the most appropriate treatment for the patient, between CHM and CT.

Among them, frailty has become increasingly recognized as one of the most important issues in elderly cancer patients who are receiving conventional antitumor treatment [32, 33]. Frailty is closely related to performance status and higher incidence of bone marrow suppression [34]. Thus, the presentation of frailty in patients contributes to the treatment dilemma involved in the use of CT. The increased risk of undertreatment is indicated in the present study, which found in the CHM group a high proportion of patients more than 70 years old who previously had received conventional therapy and rejected CT. Our previous study and the study of Wang et al. suggest that, compared to patients who received CT, patients who received CHM had a higher potential of maintaining performance status and lower risk of adverse events, such as bone marrow suppression which was closely associated with frailty [24, 35]. Based on the results of the stratified analysis in this present study we found that patients those previously treated probably gain more survival benefit from CHM compared to CT (HR:0.83, 95% CI:0.68 -1.01). CHM might be a treatment choice that can be used in patients with frailty and high possibility of side effects after CT.

In summary, we found no significant difference in survival of elderly patients with advanced NSCLC and KPS ≥ 60 between the CHM and CT groups. The possibility of noninferior efficacy and lower incidence of side effects made CHM possible to be an alternative therapy for elderly patients with advanced NSCLC. Especially, CHM might be an alternative therapy for previously treated elderly patients with EGFR wild type advanced NSCLC. In fact, these patients are the most prone to discontinue CT due to the severe side effects of CT, posing a treatment dilemma for clinicians between actions of overtreatment and undertreatment. Thus, when clinicians encounter patients with these characteristics in clinical practice, CHM as a therapy might be recommended. More studies are warranted to investigate these possibilities.

The implication of the study is that we found the possibility of noninferior efficacy of CHM and A subgroup which might receive greater benefit from CHM is identified. Based on the findings, a noninferiority RCT study on survival of elderly patients with EGFR wild type advanced NSCLC between CHM and CT as well examining side effects as a secondary superiority endpoint is warranted.

Limitations of the current study include the potential for selection bias and residual confounding based on unmeasured factors that cannot be excluded. Firstly, although we used statistical techniques to mitigate the potential imbalance between the treatment groups based on measured prognostic factors, some factors adjusted as confounders in other studies, such as lung function and modified Charlson comorbidity index (CCI), were not included in this study. The correlation between CCI and risk of death is controversial [36]. However, even if CCI has a negative impact on the prognosis, the effect of CHM on improving the prognosis would be underestimated as patients are prone to receive CHM and reject CT, when the patients' CCI is already high. For the effect of lung function on the result, the situation is similar with that of CCI. Secondly, patients who were heavily pretreated with chemotherapy were more represented in the Chinese herbal medicine group. This could be apparently seen as an imbalance favoring the chemotherapy group. However, on the contrary, an opposite bias could occur, because patients with a longer history of treatment and still fit could reasonably be characterized by a more indolent disease. This might also weak the evidence. Thirdly, all the included studies were conducted by the current research team, limiting the generalization of the results. These should be addressed by confirmatory studies by other research groups. Fourthly, sample size might be not adequate to guarantee enough power for clinically relevant difference, limiting its evidence.

## 5. Conclusion

Survival may be anything from 13% better to 10% worse in the CHM group indicate that the possibility of noninferior efficacy of CHM. There is a trend that previously treated elderly patients with EGFR wild type advanced NSCLC might gain more survival benefit from CHM. Thus, CHM probably be an alternative therapy for elderly patients with advanced NSCLC. However, limited by the design and unpreplanned study hypothesis, the results must be confirmed by randomized control trial before making a definite conclusion.

## Figures and Tables

**Table 1:** Key characteristics of three studies.

Study design	Patients	Intervention and Control	Outcomes	Enrollment Period	Follow up End
RCT [22]	(1) Advanced NSCLC; (2) EGFR wild type or untested; (3) Age *⩾* 18 years old; (4) KPS *⩾* 60; (5) Suitable for CT; (6) Expected life span of three months or more; (7) Previous treatment should be stopped for at least 1 month.	CHM vs CHM +CT vs CT	Overall Survival	30/12/2001 - 23/07/2003	31/01/2004

Prospective Cohort Study [23]	(1) Advanced NSCLC; (2) EGFR wild type or untested; (3) Age *⩾* 60 years old; (4) KPS *⩾* 60; (5) Previous treatment should be stopped for at least 1 month.	CHM vs CT or BSC	Overall Survival	12/03/2008 - 29/06/2010	31/12/2010

Retrospective Cohort Study [21]	(1) Advanced NSCLC; (2) EGFR wild type or untested; (3) Age *⩾* 18 years old; (4) KPS *⩾* 60; (5) All initial treatment; (6) Patients received EGFR tyrosine kinase inhibitors excluded. (7) The duration of treatment patients received should be at least 1.5 months.	CHM vs CHM +CT vs CT	Overall Survival	12/08/2010 - 20/04/2016	31/10/2016.

Note: NSCLC, non-small-cell lung cancer; EGFR, epidermal growth factor receptor; KPS, Karnofsky performance scores; CHM, Chinese herbal medicine; CT, chemotherapy; BSC, best supportive care; RCT, randomized controlled trial.

**Table 2:** Patient characteristics at baseline.

	**CT**	**CHM**	**P-value**
	**(n=224)**	**(n=262)**	
Gender			0.001
Male	171 (76.3%)	164 (62.6%)	
Female	53 (23.6%)	98 (37.4%)	
Age (continuous)			0.002
Mean ± SD	67.9 ± 5.6	69.6 ± 5.9	
Age (categorical)			0.001
⩽70	154 (68.8%)	143 (54.6%)	
>70	70 (31.2%)	119 (45.2%)	
Smoking Status			0.087
Missing	3 (1.3%)	0 (0.0%)	
Non-smokers	108 (48.2%)	142 (54.2%)	
Smokers	113 (50.4%)	120 (45.8%)	
Tumor Location			0.535
Missing	42 (18.8%)	45 (17.2%)	
Central Type	59 (26.3%)	81 (30.9%)	
Peripheral Type	123(54.9%)	136(51.9%)	
Pathology			0.537
Non-SQCC	104 (46.4%)	129 (49.2%)	
SQCC	120 (53.6%)	133 (50.8%)	
Stage			0.082
III	67 (29.9%)	98 (37.4%)	
IV	157 (70.1%)	164 (62.6%)	
Previous Treatment			0.014
No	150 (66.8%)	147 (56.1%)	
Yes	74 (33.0%)	115 (43.9%)	
KPS			0.216
60	20 (8.9%)	32 (12.2%)	
70	61 (27.2%)	56 (21.4%)	
*⩾*80	143 (63.8%)	174 (66.4%)	
Clinical Study			0.655
1	50 (22.3%)	65 (24.8%)	
2	131 (58.5%)	154 (58.8%)	
3	43 (19.2%)	43 (16.4%)	
Follow Up Period			
Median (95% CI)	550 (448, 599)	450(384, 561)	0.705^a^
Status			0.629
Alive	55 (24.6%)	65 (24.8%)	
Died	146 (65.2%)	163 (62.2%)	
Lost to Follow Up	23 (10.3%)	34 (13.0%)	

Note: SQCC, squamous cell carcinoma; KPS, Karnofsky performance scores; CHM, Chinese herbal medicine; CT, chemotherapy; SD, standard deviations. a: log rank test.

**Table 3:** Hazard ratio estimates based on stratified cox model.

	**Univariate Analysis**		**Multivariate Analysis**	
	**HR (95**%** CI)**	**p-value**	**HR (95**%** CI)**	**p-value**
Gender				
Male	1.00			
Female	0.8117 (0.68, 1.)	0.261		
Age (continuous)	1.00 (0.99, 1.02)	0.664		
Age (categorical)				
⩽70	1.00			
>70	1.08 (0.86, 1.37)	0.491		
Smoking Status				
Non-smoker	1.00		1.00	
Smokers	1.20 (0.96, 1.51)	0.112	1.15 (0.84, 1.46)	0.221
Tumor Location				
Central Type	1.00			
Peripheral Type	1.05 (0.81, 1.36)	0.726		
Pathology				
Non-SQCC	1.00			
SQCC	1.09 (0.86, 1.38)	0.467		
Stage				
III	1.00		1.00	
IV	1.13 (1.00, 1.28)	0.054	1.11 (0.97,1.26)	0.103
Previous Treatment				
No	1.00			
Yes	1.06 (0.82, 1.37)	0.671	1.03 (0.78, 1.34)	0.853
KPS				
60	1.00		1.00	
70	0.70 (0.46, 1.07)	0.096	0.74 (0.48, 1.14)	0.168
*⩾*80	0.36 (0.23, 0.56)	<0.001	0.39(0.25, 0.61)	<0.001
Treatment				
Chemotherapy	1.00		1.00	
CHM	0.97 (0.87, 1.09)	0.588	0.98 (0.87, 1.10)	0.751

SQCC, squamous cell carcinoma; KPS, Karnofsky performance scores; CHM, Chinese herbal medicine; HR, hazard ratio; CI, confidence intervals.

**Table 4:** Hazard ratios for relative survival of CHM and CT groups in selected subgroups.

	Unadjusted HR (95% CI)	p-value	Adjusted HR (95% CI)	p-value
Pathology ^a^				
Non-SQCC	1.01 (0.86,1.19)	0.906	1.01(0.85, 1.19)	0.918
SQCC	0.97 (0.83,1.14)	0.736	1.02 (0.86, 1.21)	0.792
Stage ^b^				
III	0.91 (0.74,1.12)	0.357	0.92 (0.74,1.14)	0.429
IV	1.01 (0.88,1.16)	0.859	0.99 (0.86,1.15)	0.928
Smoking Status ^c^				
Non-smokers	0.95 (0.81,1.12)	0.563	0.97 (0.82,1.15)	0.720
Smokers	1.02 (0.98,1.20)	0.802	1.01 (0.86,1.20)	0.876
KPS ^d^				
60-70	0.91 (0.76, 1.10)	0.360	0.92 (0.76, 1.11)	0.364
*⩾*80	1.02 (0.88, 1.17)	0.835	1.07 (0.92 1.25)	0.368
Age ^a^				
60-70	0.96 (0.83, 1.11)	0.568	0.97 (0.83, 1.13)	0.669
>70	0.97 (0.80, 1.17)	0.732	0.98 (0.81, 1.19)	0.857
Previous Treatment ^e^				
No	1.07 (0.93, 1.24)	0.338	1.08 (0.94, 1.25)	0.290
Yes	0.80 (0.66, 0.97)	0.022	0.83 (0.68, 1.01)	0.063

Note: a: stratified by studies and multivariate Cox model adjusted for smoking status, KPS, stage, and previous treatment; b: stratified by studies and adjusted for smoking status, pathology, and previous treatment; c: stratified by studies and adjusted for stage, KPS, and previous treatment; d: stratified by studies and adjusted for smoking status, stage, and previous treatment; e: stratified by studies and adjusted for smoking status, stage, and KPS. SQCC, squamous cell carcinoma; KPS, Karnofsky performance scores; CHM, Chinese herbal medicine; CT, chemotherapy; HR, hazard ratio; CI, confidence intervals.

## Data Availability

The raw and processed data used to support the findings of this study are available from the corresponding author upon request.
